# Exploring the relationship between the big five personality characteristics and dietary habits among students in a Ghanaian University

**DOI:** 10.1186/s40359-019-0286-z

**Published:** 2019-02-22

**Authors:** Freda Dzifa Intiful, Emefa Gifty Oddam, Irene Kretchy, Joana Quampah

**Affiliations:** 1 School of Biomedical and Allied Health Sciences, Department of Dietetics, University of Ghana, P.O. Box KB 143, Korle- Bu, Accra, Ghana; 2 School of Pharmacy, Department of Pharmacy Practice and Clinical Pharmacy, University of Ghana, Accra, Ghana

**Keywords:** Dietary, Extraversion, Conscientiousness, Neuroticism, Agreeableness and openness, Students, Ghana

## Abstract

**Background:**

Adherence to good dietary practices has been linked to disease prevention and better quality of life yet, University students are known to have poor dietary intake and diet quality. For an effective behaviour modification in dietary habits, an understanding of the association between an individual’s personality traits and dietary habits are of much significance.

The aim of this study was to determine the relationship between personality traits and dietary habits among University students in Ghana.

**Methods:**

A cross-sectional design involving 400 students was employed. Information on socio-demographic characteristics and Body Mass Index were obtained. The big 5 Personality traits (extraversion, conscientiousness, agreeableness, openness and neuroticism) was assessed using a 50-item International Personality Item Pool (IPIP) by Goldberg. The three factor eating questionnaire (TFEQ) was used to obtain further information on dietary habits.

**Results:**

The majority of the students had high scores for conscientiousness. Except for neuroticism, all the personality traits had a significant association with at least one of the dietary habits explored. Extraversion was positively associated with neophagia (*p* = 0.028) and food interest (*p* = 0.008), conscientiousness was associated with variety (*p* = 0.045) and sugar moderation (*p* = 0.006), agreeableness was associated with neophagia (*p* = 0.005), skipping of meals (*p* = 0.007) and variety (p = 0.005) and openness associated with food interest (*p* = 0.009).

**Conclusion:**

Personality traits showed associations with certain dietary habits but further studies are required to identify persons who are at risk of diet related diseases to inform the development of appropriate interventions.

## Background

Dietary habits refer to the set of choices or decisions one makes with regards to foods eaten. They involve what to eat, when to eat, how much to eat and where to eat [1]. These are affected by the taste preferences, variety in foods selected, frequency of meal consumption, portion sizes, snacking behaviour and skipping of meals. College students are known to have poor dietary intakes and diet quality. Several factors such as changes in residence, time management or convenience, eating out, financial constraints, family influence, obsession to control weight and nutrition misconception have been associated with this trend of poor dietary habits [2, 3].

There still remains a gap between dietary knowledge and actual dietary intake within the population such that people still find it difficult to change from negative dietary patterns to healthy options [4]. It has been postulated that the complex interaction between psychological, cultural, environmental and behavioural factors exerts an influence on an individual’s ability to alter dietary habits [5].

Understanding of the association between an individual’s personality traits and food habits have been posited to be relevant for an effective behaviour modification in eating habits [6]. Some studies have shown an association between eating disorders, body weight and personality. One of such studies found dietary disinhibition to be strongly associated with adult weight gain. Furthermore, dietary restraints were reported to reduce this effect when dietary disinhibition was high [7]. Provencher and colleagues also observed various psychological factors to be associated with personality traits and some eating behaviours [8].

Personality traits are behavioral characteristics that are consistently expressed by a person or the distinct patterns exhibited in behaviour [9]. Contemporary personality psychologists widely agree that there are five core domains or dimensions of traits that interact to form personality and shape social landscape [10]. These personality traits are also known as the ‘Big 5’ or the ‘Five Factor Model’ personality traits [11]. The ‘Five Factor Model’ has been shown to account for different traits in personality without overlapping with other traits and has demonstrated consistency in interviews, self-descriptions and physical observations [12]. These traits are broadly categorized as extraversion, agreeableness, conscientiousness, neuroticism and openness to experience [12, 13]. Certain personality traits have been linked to weight among children. For instance, low conscientiousness and high impulsivity have been associated with high body mass index and unhealthy food choices among children [1, 14]. Furthermore, a positive correlation was observed between psychoticism and unhealthy eating and neuroticism with pickiness and neophobia whereas neuroticism was negatively correlated with healthy eating and health habits [1].

The relationship between psychological factors such as one’s emotions or personality trait and how they determine eating habits may still be relevant. In a review, Macht could argue the relationship between emotions and eating habits, positing that emotions could regulate eating, likewise eating may regulate one’s emotions [15]. Also in a more recent article, authors were able to establish that the big five personality traits could be a useful tool in identifying people that may have challenges living with food allergies [16].

Knowledge of the influence of personality trait on dietary habit is of relevance in health promotion and individualizing dietary health care plan and not a one size fits all approach [8, 17]. Individualized interventions take into account demographic characteristics such as sex and age, culture and beliefs, eating practices and in rare circumstances, personality attributes [18]. Some studies have examined the links between personality attributes and eating practices among adolescents and college students [1, 18, 19]. Also, the interaction between gender, personality traits and dietary habits is under explored particular in Africa. Elsewhere, some studies have shown males exhibiting poor dietary habits compared to females [20, 21]. There is also evidence establishing the interaction between personality traits, gender and eating habits. Among Norwergian children, girls with lower conscientiousness and higher neurotism were more likely to consume sweet drinks [12]. Therefore researching into personality traits of college students may provide more insight into the determinants of diet quality [19]. However research in the area of personality traits and diet is highly under explored in Ghana. This study therefore aimed at determining the relationship between personality traits and dietary habits among college students in a Ghanaian tertiary academic institution.

## Methods

### Study design and area

The study was a descriptive school-based cross-sectional study conducted among college students at the University of Ghana.

### Study participants and sample size

The study included undergraduate students of the University of Ghana who consented to be part of the study. Respondents included both resident and non-residential students. Pregnant women and students whose anthropometric measurements could not be taken easily were excluded from the study. Based on a 95% Confidence interval, precision of 5, 50% probability and an attrition rate of 4%, a sample size of 403 was obtained but eventually 400 participants consented to be part of the study.

### Sampling

The sampling followed a multistage approach. Students were recruited from the four main colleges in the University i.e. the Colleges of Health, Education, Humanities, and Basic and Applied Sciences. Balloting was used to select two colleges out the total of four. Eight departments were further selected through the same process of balloting from the two schools earlier selected. Students in the departments were randomly selected and then approached to be part of the study.

### Ethical consideration

The study was approved by the School of Biomedical and Allied Health Sciences Ethics and Protocol Review Committee with the code number SBAHS-ET./10,443,580/AA/6A/2012–2013. A written informed consent was obtained from each participant before data collection.

### Measurements

Data were obtained using a structured, self-administered questionnaire. Data gathered included socio-demography, body weight, height and Body Mass Index (BMI), following standard procedures. Personality traits were assessed using a 50- item International Personality Item Pool –(IPIP) tool [22]. The 50-item IPIP tool measures the markers of the big five factor structure reported by Goldberg [23]. A “Three-Factor Eating Questionnaire” (TFEQ) was used to assess three aspects of the dietary habits which are Cognitive restraint of food intake involving monitoring and control of food intake and body weight; Disinhibition of control of eating which involves tendency to continue eating even when satiated, hunger or emotional eating i.e. finding solace in food when stressed or reaction to external cues [24, 25]. The TFEQ comprised of 18 items. Nine (9) of the items focused the control of food intake and body weight, six (6) concerned with disinhibition of control of eating and the remaining three (3) on emotional eating. Participants had to choose among four responses which appropriately relates to them most. Each of the four (4) responses followed a scoring system. The total score ranged between 18 and 72. The higher the score the more depended upon a particular dietary behaviour.

Additional dietary information was taken using a structured questionnaire which assessed pickiness (being fussy, choosy or selective with regards to food), ‘neophagia’ (acceptance of new and unusual foods such as foods from other cultures), food interest (having strong liking for food as compared to one who finds having to eat as a bother and would only eat because he/she has to eat), variety seeking, skipping of meals, consumption of fiber, consumption of fruits and vegetables as well as intake of fats, sugar and salt. Students were asked to indicate the strength of their agreement with specific statements pertaining to the dietary habits on a true or false scale which was expanded to definitely true, mostly true, mostly false and definitely false. False for a reversed question was taken as true (compare ‘I like to stick to the foods that I know’ to ‘I enjoy trying new foods’). A number of statements were analyzed (based on this true or false scale) to determine whether or not the respondent was prone to the dietary habit in question.

The International Personality Item Pool (IPIP) is a validated tool for assessing the personality domains of conscientiousness, openness, neuroticism, extraversion and agreeableness [17]. This comprehensive 50-item self-assessment personality test instrument measures the strength of these five fundamental dimensions of personality. Respondents were given a list of statements concerning their perception of themselves in a variety of situations and were to choose from a scale answers that most closely reflected their attitude by indicating the strength of their agreement with each statement. Although not indicated on the actual survey questionnaire, there were numbers in parentheses after each IPIP scale item indicating the type of personality factor being measured, i.e. (1) Extraversion, (2) Agreeableness, (3) Conscientiousness, (4) Emotional Stability, and (5) Openness, as well as the direction of scoring the scale of 1–5 (i.e. positive or negative). The negatively keyed items were reverse scored. A sum of all the values of the scale items was obtained to give the total scale score once the numbers were assigned for all of the items. The individual’s personality traits of extraversion, openness, neuroticism and conscientiousness were then calculated based on their responses. An individual at or above the fiftieth percentile in a particular trait was considered to be high in that trait. Scores below the fiftieth percentile were considered low. This categorization was done to clearly depict where personality strengths and weaknesses of the participants fall.

### Data analysis

Data collected were analyzed using SPSS version 20.0. Data were summarized using percentages, means and standard deviations. Chi-square analysis was used to test for association between measured dietary habits and Body Mass Index (BMI) and dietary habits and personality traits. Independent T-test was performed for differences between the scores obtained female and the male students. The level of significance was set at *p* ≤ 0.05. Cronbach’s alpha was used to test for the reliability of the questionnaires that were used.

## Results

### Demographic and BMI characteristics of students

Table 1 describes the demographic characteristics of the students. A total of 400 students participated comprising of 230 (57.5%) males and 170 (42.5%) females. The mean age of the students was 21.19 ± 1.96 years. The majority of the students were in the 2nd and 3rd years. The majority of the respondents were within the normal range of BMI (72.8%). About 15% of them were overweight or obese. However more females were overweight (17.8%) or obese (4.1%) than their male counterparts (8.3 and 2.6% respectively). An independent t-test performed showed no significant differences in the BMI’s between the males and females.

**Table 1:** Demographic characteristics of respondents (*N* = 400)

Variables	Male (230) *n* (%)	Female (170) *n* (%)	Total N (%)	*P*-value
Age (Mean ± SD) years	21.50 ± 2.04	20.76 ± 1.77	21.19 ± 1.96	0.008*
Year of study				
1	50 (21.7)	38 (22.4)	88 (22.0)	
2	63 (27.0)	47 (27.6)	110 (27.5)	
3	55 (23.9)	54 (31.2)	109 (27.3)	
4	62 (26.9)	31 (18.2)	93 (23.3)	
BMI (kg/m^2^) Mean ± SD	21.80 ± 3.28	22.41 ± 3.63	22.06 ± 3.44	0.05
Underweight	29 (12.7)	17 (10.1)	46 (11.5)	
Normal	175 (76.1)	115 (68.0)	291 (72.8)	
Overweight	19 (8.3)	30 (17.8)	49 (12.3)	
Obese	6 (2.6)	7 (4.1)	13 (3.3)	

**Fig. 1:**
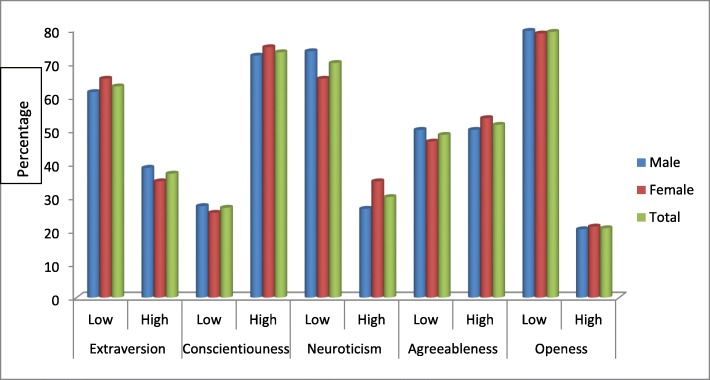
Personality trait of students

Assessment of their personality traits showed that more than half of the respondents had high scores for conscientiousness (73.2%) and agreeableness (51.5%) and low scores for extraversion (63%), neuroticism (70%) and openness (79.3%). Both males and females showed similar trends with their personality traits. (Fig. 1).

### Reliability statistics of tests

The subscales for measuring personality traits, each consisting of 10 items were found to be reliable (extraversion α =0.712, agreeableness α =0.698, openness α = 0.738, neuroticism α = 0.740, conscientiousness α =0.724). Cronbach’s alpha for three (3) items under each of the subscales for dietary habits were 0.754 (cognitive restraint), 0.740 (disinhibition) and 0.780 (emotional eating). The rest ranged between 0.668 and 0.782.

### Dietary habit traits of students

Table 2 shows the percentage of students exhibiting the various dietary traits and the *P*-values obtained after conducting an independent T-test for differences between the scores obtained by the females and the males. Significant differences were observed between the males and females in the areas of emotional eating, pickiness, neophagia, fiber consumption, sugar and salt moderation. A significant proportion of females than males reported being emotional eaters, picky eaters, practicing neophagia and consuming fiber-rich foods. The proportion of male respondents reporting moderate sugar intake was significantly higher than the proportion observed in females. No differences were observed with salt intake between males and females.

**Table 2:** Table showing the dietary habits of students according to gender

	Total	Males	Females	*P* – value
Disinhibition	19.0	20.9	16.5	0.635
Cognitive Restraint	25.2	21.7	30	0.060
Emotional eating	19.5	15.7	24.7	0.024*
Pickiness	29.0	24.8	34.7	0.031*
Neophagia	33.8	28.7	40.6	0.013*
Food interest	47.5	47.4	47.6	0.960
Variety	43.8	42.6	45.3	0.593
Skipped meals	44.0	43	45.3	0.654
Fiber consumption	23.2	19.1	28.8	0.023*
Fruits and vegetables	58.2	56.5	60.5	0.415
Fats moderation	45.8	44.3	47.6	0.513
Sugar moderation	36.2	42.4	31.7	0.029*
Salt moderation	45.5	49.6	40	0.058

### Relationship between personality traits, dietary habits and BMI

The association between the personality traits, dietary habits and BMI of the students are shown on Tables 3 and 4. No significant association was observed between BMI and dietary habits, however pickiness was positively associated with waist-to-hip ratio in both males and females. In determining the association between personality trait and dietary habits, extraversion (*p* = 0.028), agreeableness (*p* = 0.045) and openness (*p* = 0.009) were all significantly linked to neophagia. Extraversion was significantly associated with food interest (*p* = 0.008), conscientiousness significantly associated with variety (p = 0.045), agreeableness was also associated with skipping meals (*p* = 0.007) and conscientiousness associated with moderate sugar intake (*p* = 0.006).

**Table 3:** Association between personality traits and dietary habits of students

	Extraversion	Conscientiousness	Neuroticism	Agreeableness	Openness
Disinhibition	0.198	0.702	0.541	0.135	0.699
Cognitive Restraint	0.531	0.996	0.353	0.359	0.579
Emotional eating	0.455	0.805	0.205	0.768	0.800
Pickiness	0.177	0.323	0.665	0.870	0.985
Neophagia	0.028*	0.243	0.908	0.045*	0.009*
Food Interest	0.008*	0.968	0.827	0.528	0.887
Variety	0.273	0.045*	0.582	0.005*	0.675
Skipping meals	0.220	0.066	0.629	0.007*	0.713
Fiber	0.171	0.764	0.588	0.092	0.780
Fruits	0.965	0.126	0.177	0.310	0.679
Fat moderation	0.634	0.642	0.196	0.310	0.213
Sugar moderation	0.249	0.006*	0.734	0.248	0.780
Salt moderation	0.367	0.197	0.148	0.957	0.850

**Table 4:** Association between dietary habits and BMI

Dietary Habits	BMI P-values
Disinhibition	0.918
Conscientiousness	0.382
Emotional eating	0.686
Pickiness	0.941
Neophagia	0.676
Food Interest	0.200
Variety	0.847
Skipping Meals	0.825
Fibre Intake	0.501
Fruits and Vegetables	0.703
Fat moderation	0.255
Sugar moderation	0.050
Salt moderation	0.120

## Discussion

This study reports on an understudied area of nutrition research in Ghana where the relationship between an individual’s personality trait and dietary habits were investigated. The majority of the students had high scores on conscientiousness describing themselves in ways that portray self-discipline, dutifulness and planned behaviour as compared to disorderliness. Some trait characteristics for individuals having high scores for conscientiousness include being practical, thorough, neat, efficient, systematic and careful [26]. It is therefore commendable to have a high number of students exhibiting this trait. A slightly higher number of females were found to have high levels of conscientiousness in this study. This agrees with findings that this trait is exhibited more in females than males [27]. About 51.5% scored high on agreeableness in this study. This finding contradicts the findings of Cho et al. [28] where more of the respondents scored higher on agreeableness compared to the other traits. Individuals with high scores for agreeableness can be described as kind, sympathetic, trustful, cooperative and considerate [27]. The differences in scores could be attributed to the category of people in the different studies. This study was mainly among young adults whiles Cho et al. [28] focused on adolescents. Because adolescents are still mainly under parental control, it is likely that they may tend to exhibit the characteristics of agreeableness as explained earlier.

More males than females scored high on neuroticism. This also contradicts some studies [29, 30] in which female participants had high scores for neuroticism and agreeableness suggesting a more emotional and sociable personality compared to the male participants who had high scores in extraversion, conscientiousness and openness. Fischer et al. [30] also revealed that women tend to report more negative emotionality than men which in turn affect their dietary habits.

In this study, neuroticism was not significantly associated with any of the dietary habits. This is in contrast with other similar studies in which neuroticism was associated with dietary habits such as pickiness, neophobia, breakfast skipping and promotion of other unhealthy food choices [1, 29, 31]. In yet another study, it was reported that lower scores of neuroticism were associated with making healthy dietary choices [32]. It is difficult to explain in the present study why neuroticism was not significantly associated with any of the dietary habits. Conscientiousness was associated with variety and sugar moderation. In other similar studies, conscientiousness was linked to healthy eating behaviors such as avoidance of sweets, confectionaries and consumption of fruits [29. 31], regular eating time and avoidance of salty foods [33] with individuals having high scores for conscientiousness being more receptive to dietary advice and adoption of healthful practices [27, 30]. Personality traits have also been reported to correlate with dietary habits in the following descending order; conscientiousness, extraversion, agreeableness, emotional stability and openness [33]. In this study, extraversion was positively associated with food interest and neophagia. MacNicol et al., associated food interest with unhealthy eating and extraversion [1]. Extraverts are individuals who are warm and sociable and would not only stick to the foods they know but would like to try new foods from other cultures. Extraverts also have high food interest. They do not find having to eat to be a bother or only eat because they have to eat but they express much liking for food. Conscientiousness was associated with variety seeking and moderation in salt intake. This shows the students who were disciplined, industrious and dutiful also sought variety in their diet and moderated salt intake. Other reports suggest that conscientiousness was associated with fat moderation as opposed to extraversion [17]. Agreeableness was associated with neophagia, variety seeking and skipping of meals. This suggests that those who like to modify their character to suit others also like to try new foods but tend to skip meals which are not encouraged. In a contradictory finding, Kikuchi & Watanabe [29] observed desired traits such as avoidance and dislike of salty foods, desire to be healthy and avoidance of animal fat and burnt food in individuals with high scores for agreeableness [30].

This study could have been influenced by some limitations. The assessment of personality traits and dietary habits among the students were based on the individual’s own assessment of his/her self and therefore responses could be biased. Additionally reported eating habits of students could also be influenced by their purchasing power as well as other challenges such as time, availability of food on campus and the demands of their academic work.

To the best of our knowledge, this study is a new ground in Ghana. With the increase in the prevalence of chronic diseases as a result of changing dietary habits, the need to explore relevant ways to improve dietary habits is important. This calls for interventions tailored to the individual. Therefore the need to explore personality traits of individuals that affect dietary habits cannot be overemphasized. This study is therefore relevant in spite of the total dependence on respondents to be objective in answering questions about their personality traits.

## Conclusion

Personality traits have been shown to be associated with dietary habits but further studies are required to identify persons who are at risk of diet related diseases to inform the development of appropriate dietary interventions bearing in mind the personality traits they exhibit.
